# Liver transplantation in Wilson disease: a single-center experience

**DOI:** 10.1186/s13023-025-03827-9

**Published:** 2025-06-10

**Authors:** Zahra Beyzaei, Kiana Ghatei, Alireza Shamsaeefar, Kurosh Kazemi, Saman Nikeghbalian, Ali Bahador, Masoud Dehghani, Seyed-Ali Malekhosseini, Bita Geramizadeh

**Affiliations:** 1 Transplant Research Center, Shiraz University of Medical Sciences, Shiraz, Iran; 2 Department of Hepatobiliary Surgery, Abu-Ali-Sina Hospital, Shiraz University of Medical Sciences, Shiraz, Iran; 3 Department of Pathology, Medical School of Shiraz University, Shiraz University of Medical Sciences, Khalili St., Research Tower, Seventh Floor, Shiraz, Iran

**Keywords:** Wilson disease, Liver transplantation, Copper, Transplant outcomes, Living donors, Deceased donors

## Abstract

**Background:**

Wilson disease is a complex genetic disorder due to copper accumulation, mainly in the liver and brain. It is associated with severe liver disease, which is effectively cured by liver transplantation (LT). This study aimed to analyze the outcome of Wilson disease after LT from a single center in Iran.

**Methods:**

In this study, we analyzed data from Wilson patients and donors who received LT from March 2018 to December 2022 at Shiraz University of Medical Sciences, Shiraz. Long-term follow-up and post-LT outcomes for both deceased donor LT (DDLT) and living donor LT (LDLT) were measured. The Kaplan-Meier survival analysis was used to test the survival.

**Results:**

106 recipients with LT (LDLT, *n* = 22; DDLT, *n* = 84) were included (mean age of adult and pediatric: 33.1 and 10.8 years respectively; male: 58% and female 42%). The average serum ceruloplasmin and urinary copper levels improved in most patients, with values of 15.6 mg/dL and 32.3 µg per 24 h, respectively. For pediatric patients with Wilson’s disease, the survival rates at 6 months, 1 year, and 3 years were 97.0%, 96%, and 94.5%, and in adult patients achieved survival rates of 100%, 100%, and 75% at 6 months, 1 year, and 3 years, respectively.

**Conclusions:**

LT is considered as a principal therapeutic option with good long-term results in Wilson patients, even in those presenting with hepatic failure. Neurologic manifestations have been improved post-LT; however, de novo neuropsychiatric symptoms begin in some cases after successful liver transplantation.

## Introduction

Wilson disease (OMIM 277900) is a rare genetic disorder of copper metabolism, which is inherited in an autosomal recessive manner [1]. Mutations in the *ATP7B* gene lead to this disorder, which is located on chromosome 13, and encodes the copper-transporting ATPase. Due to the dysfunction of the *ATP7B* gene, the degradation of copper-free ceruloplasmin and failure of biliary copper excretion is happening [2]. Therefore, an abundance of copper in the liver, brain, lungs, kidneys, and other organs is accumulated, which can affect many organs, especially the brain or liver.

Clinical manifestation is presented as two main features including hepatic and neurologic forms. The hepatic form can be presented as an asymptomatic disorder to hepatocyte dysfunction. Histopathologic findings are steatosis, hepatitis, and fibrosis leading to cirrhosis, and acute liver failure, while the neurologic form manifests as neuropsychiatric symptoms including troubled speaking, muscle stiffness, tremors, anxiety, and personality changes with or without hepatic presentation [3, 4].

Currently, several medical therapies are applied for the control of Wilson patients; however, none of the medical therapies exactly cure the disease. The studies have indicated that early-stage medical therapies can even lead to normality as long as the disease has not progressed. However, little or no recovery occurred with medical therapy in advanced cirrhosis [5]. As a result, liver transplantation (LT) has increasingly been the logical choice [6, 7]. It is only recommended in Wilson patients who have liver cirrhosis or acute liver failure, while its use in neurological patients is still controversial. The first successful LT for Wilson patients was performed in 1969; however, there is not much information in the literature about the overall efficacy and outcome of LT in Wilson patients [8, 9].

The purpose of this study was to present the experience of LT in Wilson patients at Shiraz University of Medical Sciences, Abu-Ali Sina Hospital, along with an extensive report of follow-up and outcomes of patients.

## Methods

We conducted a retrospective analysis on 106 patients in our institutional patient database to identify the records of those with Wilson disease who underwent liver transplantation at an affiliated hospital of Shiraz University of Medical Sciences, between March 1, 2016, and December 31, 2022. Every patient fulfilled the diagnostic criteria according to the Clinical Practice Guidelines for Wilson disease [10]. All demographic data, clinical features, histopathological results, and biochemical investigations, related to pre-transplant assessment, transplant details, post-transplant complications, and survival were collected via the clinical charts of patients. In this study, chronic liver disease (CLD) was defined solely by histology-confirmed liver cirrhosis, and cases of chronic hepatitis or compensated advanced chronic liver disease (CACLD) were not included in the diagnostic criteria. For demographic and descriptive data, continuous variables were presented as mean ± standard deviation (SD) or median (range), while categorical variables were expressed as frequencies (%).To compare pre- and post-transplant values, a paired t-test was performed for normally distributed data, and McNemar’s test for categorical (presence vs. absence), while non-parametric data were analyzed using the Wilcoxon signed-rank test. We analyzed post-transplant survival using the Kaplan–Meier method to estimate survival curves. Differences between groups were assessed using the log-rank test. Additionally, to explore potential predictors of survival, we performed multivariable Cox proportional hazards regression analysis. Variables included in the model were age at transplantation, gender, donor type (deceased vs. living donor), and patient category (adult vs. pediatric). Given the small number of adult patients, a separate analysis was subsequently performed for the pediatric cohort only. Statistical significance was defined as a *P* < 0.05. Statistical analysis was conducted using SPSS 16.0 for Windows (SPSS Inc., Chicago, IL, USA). This study protocol was performed based on the ethical guidelines of the 1975 Declaration of Helsinki as approved by Shiraz University of Medical Sciences (Approval #: IR.SUMS.REC.1402.331).

## Results

### Baseline characteristics

A retrospective chart review was performed on 106 Wilson patients who had undergone LT at the affiliated hospitals of Shiraz University of Medical Sciences over 6 years. One-hundred and six adult and pediatric recipients were included in the analysis, with a mean follow-up duration of 21 ± 1.8 months, based on the LT date. The mean age of adult and pediatric recipients was 33.1 and 10.8 years, respectively. Chelation therapy was administered to all patients before transplantation. In the pediatric patients, the majority (87.9%) received Penicillamine, while a smaller percentage (9.3%) were treated with Trientine. Among adult patients, Penicillamine was the medication of choice for chelation therapy. Liver biopsies were performed in a subset of patients to support the diagnosis of Wilson disease when clinically feasible. In patients with severe coagulopathy or encephalopathy, the diagnosis was established based on clinical, biochemical, and radiological criteria, in accordance with standard practice. Following transplantation, histological evaluation of liver explants was conducted in all patients, providing confirmation of Wilson disease and assessment of liver pathology. All liver transplants were performed with ABO-compatible donors. In 4% of cases, compatibility was partial, involving subgroup matches within the ABO blood group system. Of 106 patients, 22 cases had living donor LT (LDLT), and 84 by deceased donor LT (DDLT). None of them needed a second liver transplant. All patients underwent liver transplants by a standard protocol using the piggyback, standard, left lobe, or segmental operation technique. No intraoperative complications were documented among all patients. All patients received immunosuppressive therapy, which included high-dose steroids (prednisolone), early start of Calcineurin inhibitors (tacrolimus), and in some cases mTOR inhibitors (sirolimus). Recipients, donor demographics, and clinical characteristics are summarized in Table 1.

**Table 1:** Demographic, clinical, and transplant-related characteristics of Wilson disease patients (*n* = 106)

Variable	Overall Patients (*N* = 106)	
	Pediatric (*n* = 98)	Adult (*N* = 8)
Age of recipient, mean (IQR, year)	10.8 (1–18)	33.1 (19–53)
Age of donor, mean (IQR, year)	9.9 (1–18)	34.1 (19–74)
Follow-up time, mean month (SD)	21 ± 1.8	21 ± 1.8
Sex (male %)	58	50
BMI median, (IQR)	23.4 (14.7–35.8)	25 (18.7–35.2)
PELD/ MELD, median (IQR)	18 (6–40)	22 (7–39)
Onset Clinical presentation, n (%)		
Hepatic Neurological Hepatic + Neurological Psychiatric	93(95.5) 0 5 (4.5) 0	6 (75) 0 2 (25) 0
Chelation therapy, n (%)		
Penicillamine Trientine	88 (89.7) 10 (9.3)	8 (100) 0
Explant liver histology, n (%)		
Cirrhosis Fibrosis No change	96 (98) 1 (1) 1 (1)	8 (100) - -
Indication for LT, n (%)		
Acute liver failure (ALF) Chronic liver disease (CLD) Progressive neurological deterioration	53 (54.1) 43 (43.8) 2 (1.1)	6 (75) 2 (25) -
ABO blood type, n (%)		
Compatible Partial incompatible	97 (99) 1 (1)	6 (75) 2 (25)
Type of donor, n (%)		
Deceased donor Living donor	78 (79.6) 20 (20.4)	6 (75) 2 (25)
Mortality, n (%)		
Within 6 Months Within 1 year Within 3 years	11 (11.2) 6 (6.1) 3 (3.1) 2 (2.0)	5 (62.5) 3 (37.5) 1 (12.5) 1 (12.5)
Graft failure type, n (%)		
Acute rejection Chronic rejection	7 (93) 6 (94)	2(25) 1 (87.5)
Surgery technique, n (%)		
Piggy-back Standard Left lobe Segmental	69 (70) 27 (27.5) 1 (1) 1 (1)	7 (87.5) - 1 (12.5) -
Immunosuppressant,		
Steroid ≥ 1 month Calcineurin inhibitor mTOR inhibitor	98 98 8	8 8 1

Values are presented as mean ± standard deviation or number (%). Post-transplant data represent values recorded at the last available follow-up for each patient

BMI, body mass index; IQR: inter-quartile range; LDLT, living donor liver transplantation; DDLT, deceased donor liver transplantation; MELD, the model for end-stage liver disease; PELD, Pediatric End-Stage Liver Disease; mTOR, mammalian target of rapamycin

### Preoperative clinical characteristics

A definite diagnosis of Wilson disease was made in all 106 patients based on a broad combination of laboratory tests, liver biopsy and clinical features including 24 h urine copper, a severe coagulopathy, serum level of ceruloplasmin, Kayser-Fleischer rings (KF rings), presence of *ATP7B* gene mutation, and neurological symptoms. In this context, “severe coagulopathy” referred primarily to liver-related coagulopathy associated with chronic advanced liver disease or cirrhosis due to Wilson disease. While hemolytic anemia is a recognized manifestation of copper toxicity, it was not specifically used as a diagnostic criterion for Wilson disease in this study. All the patients presented with a decreased level of serum ceruloplasmin (mean: pediatrics, 1.37 ± 2.1; adult, 1.32 ± 6.1) and an increase of 24-h urinary copper excretion (mean: pediatrics, 225.9 ± 0.9; adult, 276.1 ± 3.6). The KF rings were observed in 40.6% of pediatric and 25% of adult patients. In most of the patients, alanine aminotransferase (ALT) activity was elevated moderately if at all raised (range 14–682 U/L); the same was the result for aspartate aminotransferase (AST) activity (range 17–980 U/L). The mean of WBC and PLT was lower than average. However, prothrombin time (PT), INR, and total bilirubin were high in pediatrics (18.9 ± 0.9, 1.45 ± 0.1, and 4.4 ± 0.3, respectively) and adult patients (19.7 ± 1.3, 1.50 ± 0.1, and 2.2 ± 0.3, respectively). The onset presentation in 98% of patients was hepatic in nature. In 2% of patients, primarily adults, a neurological presentation was observed, which was combined with the hepatic form and included symptoms such as rigidity and ataxia in varying combinations. The detailed onset clinical presentations were as follows: 98 (100%) hepatic, 2 (25%) neurological and hepatic features (observed only in adults). The most common indication of LT in the pediatric and adult Wilson patients was acute liver failure with 54.1% and 75% respectively. The critical laboratory data are listed in Table 2.

**Table 2:** Key laboratory and clinical findings for Wilson patients (*n* = 106) pre- and post-transplant

Variables	Pre-transplant	Post-transplant**	*P* Value	Reference range
Hb (g/dL)				
Pediatric Adult	10.3 ± 1.1 11.7 ± 2.8	13.4 ± 1.8 15.4 ± 2.9	< 0.0001 < 0.0001	Children: 11.0 to 16.0 Adults (Men): 13.8 to 17.2 Adults (Women): 12.1 to 15.1
WBC count (per µl)				
Pediatric Adult	3526 ± 1.6 4620 ± 3.6	5540 ± 1.1 8254 ± 3.1	< 0.0001 < 0.0001	Children: 6,000 to 17,000 Adults: 4,000 to 11,000
PLT count (per µl)				
Pediatric Adult	91,000 ± 16 75,000 ± 26	354,000 ± 12 454,000 ± 29	< 0.0001 < 0.0001	Children: 150,000 to 450,000 Adults: 150,000 to 450,000
AST (IU/L)				
Pediatric Adult	108.4 ± 1.4 188.9 ± 3.2	53.1 ± 1.2 72.7 ± 4.4	< 0.0001 < 0.0001	Children: 10 to 50 Adults: 10 to 40
ALT (IU/L)				
Pediatric Adult	88.9 ± 2.1 124.9 ± 4.1	44.3 ± 2.9 58.9 ± 3.4	< 0.0001 < 0.0001	Children: 10 to 50 Adults: 7 to 56
BUN (mg/ dL) Pediatric Adult	10.11 ± 1.2 12.15 ± 2.7	14.5 ± 0.8 18.9 ± 2.1	< 0.0001 < 0.0001	Newborns: 3 to 12 Children (1–3 years): 5 to 18 Children (4–18 years): 5 to 20 Adult: 7–19
Alb (g/dL)				
Pediatric Adult	3.1 ± 1.0 3.3 ± 1.0	3.8 ± 0.5 5.0 ± 0.5	< 0.0001 < 0.0001	Pediatric: 3.4 to 5.4 Adult: 3.5 to 5.4
Creatinine (mg) Pediatric Adult	0.30 ± 0.3 1.05 ± 0.3	0.97 ± 0.1 0.99 ± 0.3	< 0.0001 0.87	Newborns: 0.3–1.2 Children: 0.2–0.7 Adults: 0.6–1.3
Prothrombin time (s)				Neonates (newborns): 14 to 18
Pediatric Adult	18.9 ± 0.9 19.7 ± 1.3	13.2 ± 1.2 13.4 ± 1.2	< 0.0001 < 0.0001	Infants (up to 1 year): 12 to 14 Children (1 year to adolescence): 11 to 14 Adults: 11 to 13.5
INR				
Pediatric Adult	1.45 ± 0.1 1.50 ± 0.1	1.04 ± o.1 1.18 ± o.1	< 0.0001 < 0.0001	Normal INR: 0.8 to 1.2
Total bilirubin (mg/dL)				
Pediatric Adult	4.4 ± 0.3 2.2 ± 0.3	1.8 ± 0.2 1.3 ± 0.6	< 0.0001 < 0.0001	Newborns: up to 5 mg/dL Adults: 0.1 to 1.2
Serum Ceruloplasmin (mg/dL)				
Pediatric Adult	1.37 ± 2.1 1.32 ± 6.1	13.6 ± 2.2 17.5 ± 5.1	< 0.0001 < 0.0001	Children: 18 to 50 Adults: 20 to 60
Urinary copper (µg per 24 h)				
Pediatric Adult	225.9 ± 0.9 276.1 ± 3.6	34.9 ± 7.3 30.5 ± 3.3	< 0.0001 < 0.0001	Normal range: 10 to 40 µg/24 h
Presence of KF rings, n (%)*				
Pediatric Adult	43 (40.6) 2 (25)	31 (29.2) 0	< 0.05 > 0.05	

*For KF rings, the data are categorical (presence vs. absence), and statistical analysis was performed using McNemar’s test

** Post-transplant data represent values recorded at the last available follow-up for each patient

### Postoperative main findings

Post-transplantation, the average serum ceruloplasmin level and urinary copper excretion returned to normal, with serum ceruloplasmin increasing to 15.6 mg/dL and urinary copper decreasing to 32.3 µg per 24 h after 2 months. Other factors including AST, ALT, and ALK also decreased (61.1, 49.9, and 156.3 IU/L, respectively). The mean of PT, PLT, WBC, BUN, Alb, and INR in all patients was normal. All differences between pre- and post-transplantation data in both adults and pediatric patients were highly significant, except for the creatinine level in adults (*p* = 0.87). Hence, the difference in the presence vs. absence of KF rings in adults was insignificant, possibly due to the small sample size. Established cirrhosis was observed in the majority of patients (*n* = 96, 98%) on explant liver histopathological examination (Table 2).

### Postoperative neurological outcomes

Although many patients experienced improvements in neurological manifestations post-transplantation, a subset developed new neurological symptoms or experienced a relapse of pre-existing ones. Specifically, seven patients without neurological symptoms prior to the transplant developed new symptoms, including mild encephalopathy, tremors, and dysarthria. Six months post-transplant, four patients developed mild encephalopathy, two exhibited tremors, and one developed dysarthria. Additionally, among the seven patients with pre-existing neurological symptoms, manifestations such as rigidity and ataxia improved following LT.

### Postoperative mortality and survival rates

During the four-year follow-up post-transplant, a total of 16 out of 106 patients passed away due to both hepatic and non-hepatic causes. No mortality was observed within 30 days post-transplantation. In pediatric patients, mortality occurred in 6 cases (6.1%) within 6 months, 3 cases (3.1%) within 1 year, and 2 cases (2.0%) within 3 years. In adult patients, mortality was observed in 3 cases (37.5%) 6 months, 1 case (12.5%) within 1 year, and 1 case (12.5%) within 3 years. Of those who died due to hepatic causes, 2 patients had coronary artery disease, 3 had pulmonary hypertension, and 7 experienced chronic rejection. While coronary artery disease and pulmonary hypertension are typically considered cardiovascular and pulmonary conditions, they are often associated with liver disease, particularly in patients with cirrhosis or those who have undergone liver transplantation. Coronary artery disease can be exacerbated by liver disease, and pulmonary hypertension is commonly observed in patients with advanced liver disease, often as a complication of portal hypertension. Non-hepatic causes of death included a recurrence of hepatocellular carcinoma (HCC) after liver transplantation in one patient, an infection in one, renal complications in one, and a neurological issue in one. Although HCC is a hepatic malignancy, we have categorized it as a non-hepatic cause of death because it occurred as a recurrence after the liver transplant, indicating a post-transplant malignancy rather than a direct progression of underlying liver disease.

Additionally, the role of chronic and acute rejection episodes in relation to patient mortality should be considered, as both chronic and acute rejection can contribute to graft failure and related complications, potentially leading to patient death. 9 out of 16 had episodes of acute rejection and 7 out of 16 patients had chronic rejection. It was observed that all these patients had elevated levels of AST, ALT, and urinary copper post-LT.

**Fig. 1:**
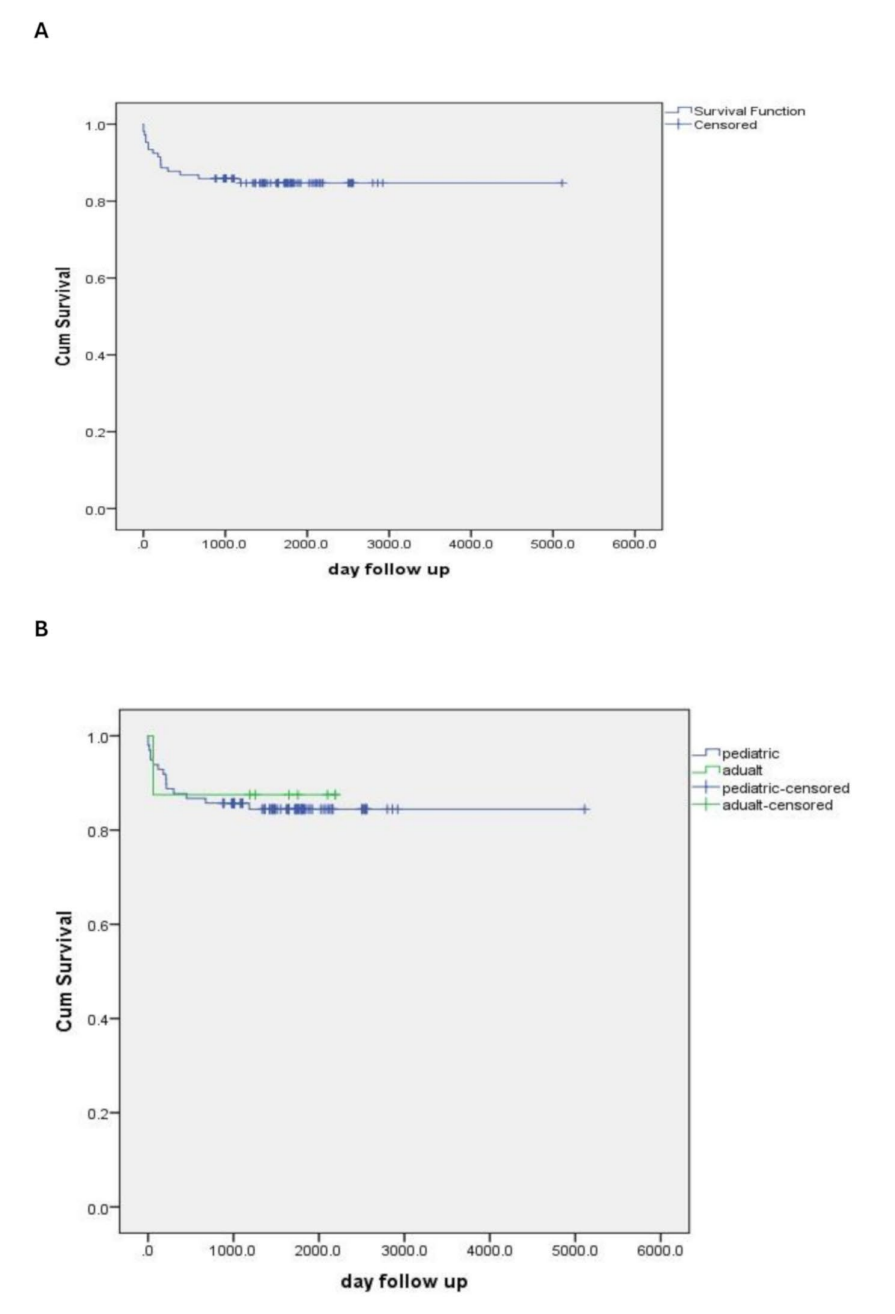
(**A**) Kaplan–Meier curve depicting overall patient survival following liver transplantation. (**B**) Kaplan–Meier survival curves stratified by patient group: pediatric patients (blue line) and adult patients (green line)

The results demonstrated a favorable prognosis for LT in patients with Wilson disease, with a survival rate exceeding 4 years based on follow-up data for patients. For pediatric patients with Wilson disease, the survival rates at 6 months, 1 year, and 3 years were 97.0%, 96%, and 94.5%, respectively. Meanwhile, adult patients with Wilson disease achieved survival rates of 100%, 100%, and 75% at 6 months, 1 year, and 3 years, respectively (Fig. 1).

In the multivariable Cox regression analysis including all patients, none of the examined variables (age at transplantation, gender, donor type, or patient category) were significantly associated with post-LT mortality (*P* > 0.05). Due to the small number of adult patients (*n* = 8), a separate Cox regression analysis was performed on the pediatric group (*n* = 98). Similarly, no baseline characteristics and PELD were identified as statistically significant predictors of survival within the pediatric cohort. These results suggest that, in our cohort, post-transplant survival was not significantly influenced by the evaluated baseline factors.

## Discussion

Liver transplantation is a cure for end-stage liver disease of Wilson disease (WD). There are different hepatic presentations in WD, so LT is performed in cirrhotic or acute hepatic failure (ALF). Based on our results, the predominant indication for LT in both pediatric and adult Wilson patients is acute liver failure. In the pediatric population, acute liver failure accounts for 54.1% of LT cases, while in the adult population, it accounts for 75%. This underscores the significance of acute liver failure as a critical factor leading to the need for liver transplantation in patients with the hepatic form of WD. Literature review and our data demonstrated that LT could partially or completely correct the underlying metabolic defects [11]. The results from our center align with findings from other global studies on LT in WD patients. In our cohort, ALF was the predominant indication for LT, which is consistent with several large-scale studies in other regions. For example, a study by Członkowska et al. reported that ALF was the primary indication for LT in WD patients, particularly in the pediatric population, with similar rates of transplantation due to acute liver failure (61%) [12]. In comparison, a report from India by Ghosh et al. indicated a slightly higher proportion of LT cases for ALF (78%), particularly in younger patients, highlighting regional differences in the presentation of WD [13].

Copper metabolism is rapidly normalized after LT, although its restoration in extrahepatic organs, such as the central nervous system and kidneys, is more gradual [14]. Serum ceruloplasmin levels typically normalize within the first month post-LT, and more than 60% of patients show complete clearing of KF rings. However, it is important to note that LT does not always restore copper metabolism to normal levels in all cases. In our study, some patients continued to have urinary copper levels above 50 µg per 24 h and persistently low serum ceruloplasmin levels. We speculate that this could be due to the effects of immunosuppressive therapy, which may alter copper metabolism. Additionally, as ceruloplasmin is an acute-phase reactant, its levels can be overestimated during the acute phase of the disease. Once the acute condition resolves, ceruloplasmin levels may decrease, potentially contributing to the persistently low levels observed in some patient’s post-transplantation. Moreover, *ATP7B*, which is primarily expressed in the liver, is also found in other tissues, including the central nervous system and kidneys. This could contribute to ongoing neuropsychiatric symptoms despite improved copper regulation in the liver [14].

In patients with neurological Wilson disease, our data showed varying degrees of neurological improvement following liver transplantation, with all patients exhibiting at least some clinical benefit. LT has been reported to reverse neurological manifestations in many patients with Wilson disease. Therefore, timely consideration of LT is crucial, as delays may result in irreversible neurological damage [15–19]. However, this hypothesis has not been confirmed in patients undergoing LT early after the onset of neurological symptoms [1]. It is important to note that while LT generally leads to neurological improvement, there is a recognized risk of neurological deterioration following LT. This deterioration may arise due to perioperative factors, such as metabolic imbalances, immunosuppressive medication effects, or unrecognized ongoing copper toxicity. Outcomes can vary, with some patients experiencing transient worsening and others showing more prolonged deficits [20, 21]. Management strategies include optimizing perioperative care, close monitoring of copper levels, and tailoring immunosuppressive regimens. Further studies are needed to elucidate the causes and refine interventions to mitigate the risk of post-LT neurological deterioration.

Additionally, while the improvement of neurological symptoms post-LT is widely observed, our results contribute to the growing body of evidence suggesting that neurological deterioration may occur after LT in a subset of patients. Studies from the United States and Europe have similarly reported that while many patients experience improvements in neurological manifestations post-transplantation, others develop new neurological symptoms or have worsening of pre-existing ones [22, 23]. These findings underscore the need for ongoing surveillance and tailored management strategies to prevent or mitigate neurological complications post-LT.

Sixteen of the Wilson patients in this study died after LT. The deceased patients had an elevated mean of AST, ALT, and level of urinary copper after LT as well as a higher rate of acute rejection. Several death events occurred in the first year post-LT (early- rejection) similar to other reports [24–26]. Furthermore, regarding survival post-LT, our cohort’s mortality rate (16 patients out of 106) and the factors contributing to this mortality, such as elevated AST/ALT and high urinary copper levels post-transplant, are consistent with reports from centers in Europe and Asia [27, 28]. In particular, the occurrence of early post-transplantation deaths due to acute rejection is in line with studies that emphasize the critical role of early immune management post-LT in WD patients [29].

According to previous reports on Wilson patients, the combination of hepatic and neuropsychiatric symptoms is the important factor influencing survival post-LT, which decreases survival in comparison to patients with liver involvement alone [4, 30–32]. The International Wilson guidelines do not currently recommend liver transplantation as a treatment option for neurological Wilson patients [33]. However, a recent article by Litwin et al. systematically assessed all available evidence on the safety of liver transplantation in this patient population [7]. The authors concluded that liver transplantation is a promising treatment option for neurological Wilson patients, even in cases of severe symptoms. However, post-LT, the potential for worsening neurological symptoms, particularly major events such as coma, seizures, and encephalopathy, exists. According to reports, LDLT is associated with a significantly lower incidence of neurological complications when compared to patients who undergo DDLT [34, 35]. To better understand the causes and consequences of neurological symptoms in liver transplant recipients, it is essential to conduct a routine preoperative neurological evaluation and thorough post-operative examination. So, further investigations are needed to validate these findings.

Our study has several limitations, including its retrospective design, the absence of neurological data for some patients, lack of information on the resolution of KF rings post-LT, and missing post-LT data on serum ceruloplasmin levels and 24-hour urinary copper levels. While our data demonstrate clinical improvement in neurological symptoms in all patients post-LT, we did not specifically assess brain MRI findings to confirm these changes. Future studies that incorporate imaging data could offer a more comprehensive understanding of the neurological recovery process following liver transplantation.

In conclusion, this study revealed the benefit of LT for Wilson patients and good survival post-LT with improvement in metabolic manifestations in most patients. LT is typically indicated in patients with Wilson disease who present with acute liver failure or severe hepatic insufficiency unresponsive to chelation therapy—a topic that remains subject to ongoing debate. Based on our findings, it is appropriate to further explore the role of LT in patients with isolated neurological manifestations.

## Data Availability

The data generated for this study are available upon reasonable request from the corresponding author.

## Abbreviations

ALT: Alanine aminotransferase
ALF: Acute hepatic failure
AST: Aspartate aminotransferase
CLD: Chronic liver disease
CACLD: Compensated advanced chronic liver disease
DDLT: Deceased donor liver transplantation
HCC: Hepatocellular carcinoma
IS: Immune suppression
KF: Kayser-Fleischer rings
LDLT: Living donor liver transplantation
LT: Liver transplantation
PTLD: Post-transplant lymphoproliferative disorder
WD: Wilson disease
